# VALIDITY AND RELIABILITY OF THE ROWLAND UNIVERSAL DEMENTIA ASSESSMENT SCALE: IMPLICATIONS OF AN INDONESIAN STUDY

**DOI:** 10.1093/geroni/igac059.1814

**Published:** 2022-12-20

**Authors:** Renata Komalasari, Elias Mpofu

**Affiliations:** University of North Texas, Denton, Texas, United States; University of North Texas, Denton, Texas, United States

## Abstract

Standardized dementia screening self-report assessments from developing countries are increasingly adopted in developing countries. Yet the cross-cultural transportability of dementia screening tools cannot be assumed. The Southeast Asia region has one of the fast-growing geriatric populations but no locally developed or validated measures of cognitive decline among older adults. This study aimed to determine the validity and reliability of scores from the Rowland Universal Dementia Assessment Scale (RUDAS) for use in the multiethnic older adult population in Indonesia. Participants were 135 Indonesian older adults (females = 61.5%, males = 38.5%; age range = 60-82) who were patients of a geriatric nursing centre. They completed the Indonesian translation of the Rowland Universal Dementia Assessment Scale (RUDAS-Ina). Exploratory factor analysis and confirmatory factor analysis of the scores yielded two factors explaining a total of 55.13% (the first factor explained 38.18%; the second factor explained 16.96%) of the variance for the entire set of variables. The reliability of scores from the RUDAS-Ina was marginally satisfactory (Cronbach α = .61), suggesting a need for locally developed measures for use with the Indonesian geriatric population. Future studies should consider the feasibility of developing and calibrating an adapted RUDAS to the Indonesian setting, which also may be studied in other Southeast Asian countries.

